# Crystal structure of bis­{μ_2_-3-(pyridin-2-yl)-5-[(1,2,4-triazol-1-yl)meth­yl]-1,2,4-triazolato}bis­[aqua­nitrato­copper(II)] dihydrate

**DOI:** 10.1107/S2056989016003479

**Published:** 2016-03-11

**Authors:** Roman Doroschuk

**Affiliations:** a Department of Inorganic Chemistry, Taras Shevchenko National University of Kyiv, 64, Volodymyrska Str., 01033, Kyiv, Ukraine

**Keywords:** crystal structure, 1,2,4-triazole, dinuclear copper complex, hydrogen bonds

## Abstract

The structure of the dinuclear title complex, [Cu_2_(C_10_H_8_N_7_)_2_(NO_3_)_2_(H_2_O)_2_]·2H_2_O, consists of centrosymmetric dimeric units with a copper–copper separation of 4.0408 (3) Å. The Cu^II^ ions in the dimer display a distorted octa­hedral coordination geometry and are bridged by two triazole rings, forming an approximately planar Cu_2_N_4_ core (r.m.s. deviation = 0.049 Å). In the crystal, O—H⋯O, O—H⋯N and C—H⋯O hydrogen bonds and π–π inter­actions link the mol­ecules into a three-dimensional network.

## Abstract 2

The title complex is a centrosymmetric dimer with a copper–copper distance of 4.0408 (3) Å. The Cu ions in the dimer are bridged by two triazole rings and oxygen donor ligands from water mol­ecules and nitrate anions in a distorted octa­hedral coordination geometry.

## Chemical context   

The presence in the triazole ring, three donor atoms and the possibility of introducing in the heterocycle substituents of a different nature creates the conditions for target synthesis of complexes with inter­esting structures and properties. The study of this type of coordination compound is promising since, as a result, a compound can be obtained with useful physical properties such as optical, magnetic or catalytic (Soghomonian *et al.*, 1993 ▸; Blake *et al.*, 1999 ▸). Another inter­esting aspect of these compounds is the possibility of their use as functional models of enzymes such as catechol oxidase (Moliner *et al.*, 2001 ▸; Klingele *et al.*, 2009 ▸; Selmeczi *et al.*, 2003 ▸).
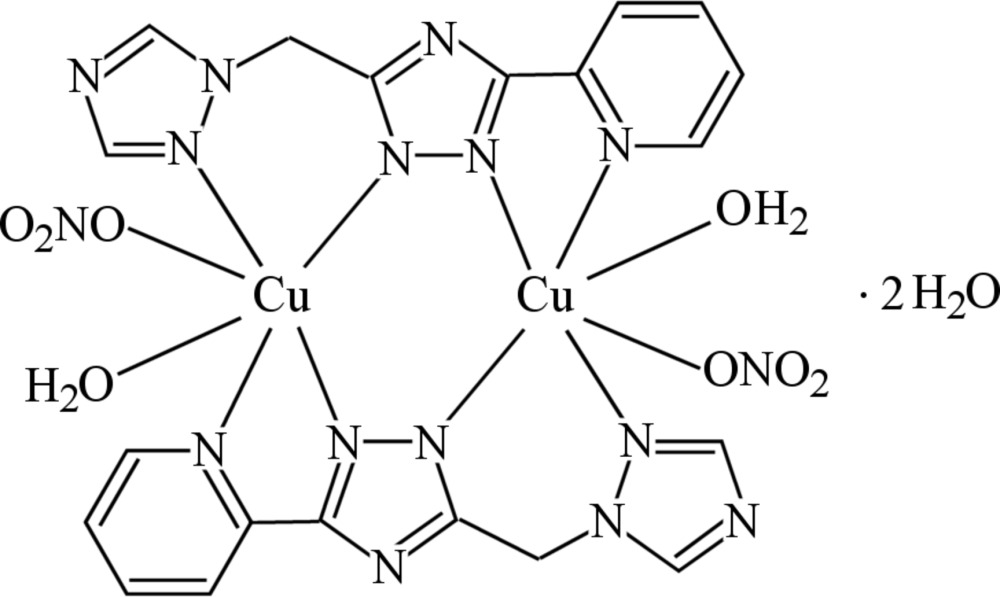


## Structural commentary   

**Figure 1:**
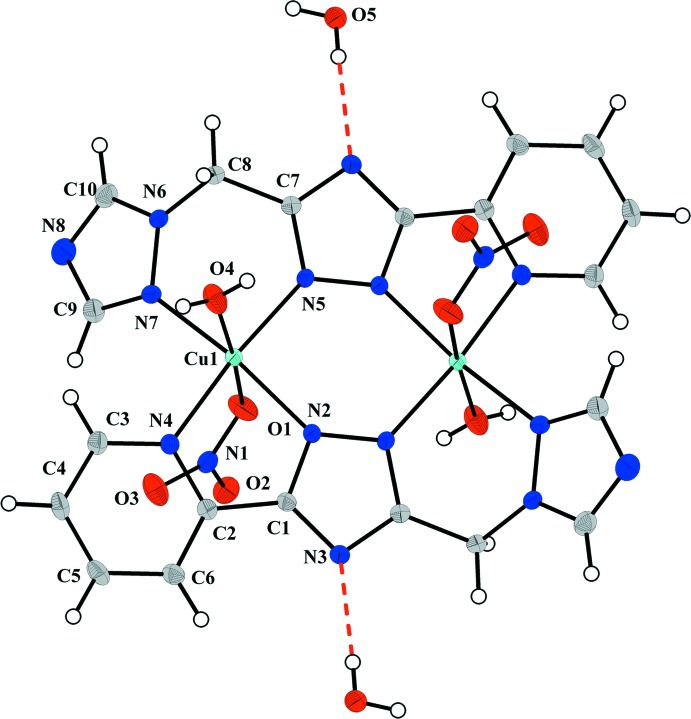
The mol­ecular structure of the title compound with displacement ellipsoids drawn at the 40% probability level. Dashed lines indicate hydrogen bonds. Unlabelled atoms are related to labelled atoms by (−*x*, 1 − *y*, −*z*).

The structure of the title complex mol­ecule (Fig. 1 ▸) has a crystallographically imposed centre of symmetry, and contains two copper(II) metal atoms doubly bridged by the triazole rings of two deprotonated ligands. Each copper(II) ion is coordinated in a distorted elongated octa­hedral geometry by one pyridine and three triazole nitro­gen atoms forming the equatorial plane, and by the O atoms of a water mol­ecule and a monodentate nitrate anion at the apices. The Cu—N bond lengths involving the bridging triazole ring [mean value 1.9722 (15) Å] are slightly, but significantly, shorter than those involving the pyridine and peripheral triazole rings [Cu1—N4 = 2.0386 (16) and Cu1—N7 = 2.0409 (17) Å]. The inner Cu_2_N_4_ core is approximately planar [r.m.s. deviation = 0.049 Å; maximum displacement 0.062 (2) Å for atom N2], with a Cu⋯Cu separation of 4.0408 (3) Å, in good agreement with the values usually observed in μ-triazolyl-bridged complexes (Haasnoot, 2000 ▸). The central triazole ring makes dihedral angles of 7.78 (8) and 49.30 (8)°, respectively, with the pyridine and peripheral triazole rings. The six-membered chelate ring Cu1/N5/C7/C8/N6/N7 assumes a boat conformation [puckering parameters: *Q*_T_ = 0.619 (2) Å; θ2 = 88.62 (16)°], while the five-membered Cu1/N2/C1/C2/N4 chelate ring adopts a flattened envelope conformation with the Cu atom as flap [puckering parameters: *Q* = 0.127 (2) Å; φ = −156.8 (8)°].

## Supra­molecular features   

**Figure 2:**
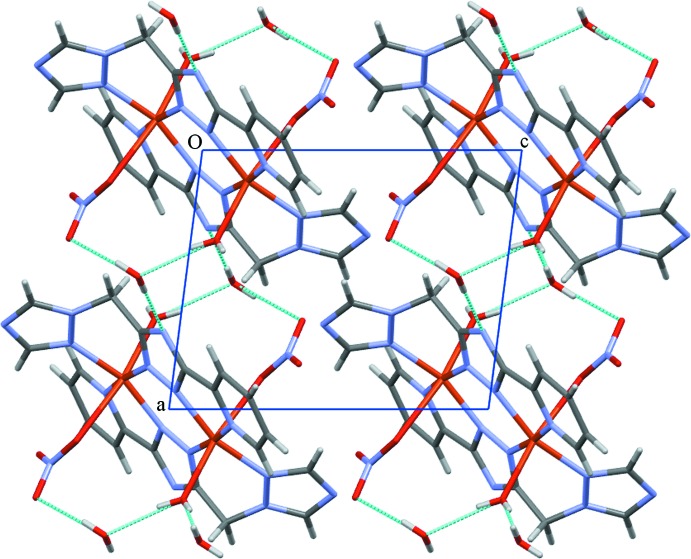
Packing diagram of the title compound, viewed along the *b* axis. Inter­molecular hydrogen bonds are shown as blue dotted lines.

In the crystal, the complex molecules and water mol­ecules of crystallization are linked through O—H⋯O, O—H⋯N and C—H⋯O hydrogen bonds (Table 1 ▸), forming a three-dimensional network (Fig. 2 ▸). The crystal structure is further stabilized by π–π stacking inter­actions with centroid–centroid separations *Cg*1⋯*Cg*2^ii^ = 3.8296 (13) Å and *Cg*3⋯*Cg*3^iii^ = 3.5372 (10), and perpendic­ular inter­planar distances *Cg*1⋯*Cg*2^ii^ = 3.5584 (9) and *Cg*3⋯*Cg*3^iii =^ 3.3234 (10) Å [*Cg*1, *Cg*2 and *Cg*3 are the centroids of the N1/C2/N3/C7^i^/N5^i^, N4/C2–C6 and N6/N7/C9/N8/C10 rings, respectively; symmetry codes: (i) −*x*, 1 − *y*, −*z*; (ii) −*x*, −*y*, −*z*; (iii) 1 − *x*, −*y*, 1 − *z*].

## Database survey   

The Cambridge Structural Database (CSD Version 5.36 with three updates; Groom & Allen, 2014 ▸), returned 45 entries with the triazole bridging fragment Cu–(N–N)_2_–Cu. The most similar are: di­aqua­bis­(μ-3,5-bis­(2-pyrid­yl)-1,2,4-triazolato-*N*′,*N* ^1^,*N* ^2^,*N*′′)bis­(tri­fluoro­methane­sulfonato-*O*)dicopper(II) (Prins *et al.*, 1985 ▸), bis­[μ-5-(pyridin-2-yl)-3-(1*H*-1,2,4-triazol-3-yl)-1,2,4-triazolato]di­aqua­dicopper diperchlorate (Zhou *et al.*, 2014 ▸), bis­[μ_3_-(pyridin-2-yl)-5-([5-(pyridin-2-yl)-1,2,4-tria­zol-1-id-3-yl]meth­yl)-1,2,4-triazol-1-ide]tri­aqua­tricopper di­perchlorate dihydrate (Gusev *et al.*, 2014 ▸) and bis­(μ-5-(2-eth­oxy-2-oxoeth­yl)-3-(pyridin-2-yl)-1*H*-1,2,4-triazol­yl)bis(acetonitrile)­bis­(perchlorato-*O*)dicopper (Khomenko *et al.*, 2012 ▸). Only 10 compounds containing a pyridyl and a methyl­ene moiety, as substituents in the 3- and 5-positions of 1,2,4-triazole, were found (Lin *et al.*, 2013 ▸; Gusev *et al.*, 2014 ▸ and references therein).

## Synthesis and crystallization   

A water solution of Cu(NO_3_)_2_·3H_2_O (0.25 mmol, 0.0605 g) was added to a hot solution of 2-[5-(1,2,4,)-triazol-1-yl-methyl-1*H*-(1,2,4)-triazol-3­yl]pyridine (0.25 mmol, 0.059 g) in water (7 ml). The transparent blue solution was left to evaporate slowly in the air and after few hours, blue single crystals suitable for X-ray analysis were obtained (yield: 67%).

## Refinement   

Crystal data, data collection and structure refinement details are summarized in Table 2 ▸. H atoms of water mol­ecules were located from a difference Fourier map and refined freely. All other H atoms were constrained to ride on their parent atoms, with C—H = 0.95–0.99 Å and with *U*_iso_(H) = 1.2*U*_eq_(C).

## Supplementary Material

Crystal structure: contains datablock(s) I. DOI: 10.1107/S2056989016003479/rz5185sup1.cif

Structure factors: contains datablock(s) I. DOI: 10.1107/S2056989016003479/rz5185Isup2.hkl

CCDC reference: 1456451

Additional supporting information:  crystallographic information; 3D view; checkCIF report

## Figures and Tables

**Table 1:** Hydrogen-bond geometry (Å, °)

*D*—H⋯*A*	*D*—H	H⋯*A*	*D*⋯*A*	*D*—H⋯*A*
O4—H41*O*⋯O5^i^	0.71 (3)	2.03 (3)	2.735 (2)	172 (3)
O4—H42*O*⋯O5^ii^	0.79 (3)	1.96 (3)	2.735 (2)	168 (3)
O5—H51*O*⋯O2^iii^	0.78 (3)	2.02 (3)	2.773 (2)	163 (3)
O5—H52*O*⋯N3^iv^	0.76 (3)	2.08 (3)	2.836 (2)	177 (3)
C5—H5⋯O1^i^	0.95	2.43	3.360 (3)	166
C8—H8*A*⋯O4	0.99	2.56	3.160 (3)	119
C8—H8*B*⋯O2^iii^	0.99	2.36	3.319 (3)	162
C9—H9⋯O3^v^	0.95	2.44	3.205 (3)	137

Symmetry codes: (i) 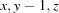; (ii) 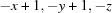; (iii) 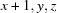; (iv) 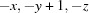; (v) 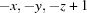.

**Table 2:** Experimental details

Crystal data	
Chemical formula	[Cu_2_(C_10_H_8_N_7_)_2_(NO_3_)_2_(H_2_O)_2_]·2H_2_O
*M*_r_	775.63
Crystal system, space group	Triclinic, *P* 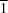
Temperature (K)	173
*a*, *b*, *c* (Å)	8.8421 (2), 8.8636 (2), 10.5686 (2)
α, β, γ (°)	70.114 (1), 88.6311 (10), 66.765 (1)
*V* (Å^3^)	709.87 (3)
*Z*	1
Radiation type	Mo *K*α
μ (mm^−1^)	1.58
Crystal size (mm)	0.50 × 0.50 × 0.45

Data collection	
Diffractometer	Bruker APEXII CCD
Absorption correction	Multi-scan (*SADABS*; Bruker, 2003 ▸)
*T*_min_, *T*_max_	0.505, 0.536
No. of measured, independent and observed [*I* > 2σ(*I*)] reflections	8672, 2945, 2711
*R*_int_	0.025
(sin θ/λ)_max_ (Å^−1^)	0.629

Refinement	
*R*[*F* ^2^ > 2σ(*F* ^2^)], *wR*(*F* ^2^), *S*	0.027, 0.073, 1.07
No. of reflections	2945
No. of parameters	233
H-atom treatment	H atoms treated by a mixture of independent and constrained refinement
Δρ_max_, Δρ_min_ (e Å^−3^)	0.26, −0.57

Computer programs: *APEX2* and *SAINT* (Bruker, 2003 ▸), *SHELXS97* and *SHELXTL* (Sheldrick, 2008 ▸) and *SHELXL2014* (Sheldrick, 2015 ▸).

## supplementary crystallographic information

### Crystal data

**Table:**

[Cu_2_(C_10_H_8_N_7_)_2_(NO_3_)_2_(H_2_O)_2_]·2H_2_O	*Z* = 1
*M**_r_* = 775.63	*F*(000) = 394
Triclinic, *P*1	*D*_x_ = 1.814 Mg m^−^^3^
*a* = 8.8421 (2) Å	Mo *K*α radiation, λ = 0.71073 Å
*b* = 8.8636 (2) Å	Cell parameters from 6116 reflections
*c* = 10.5686 (2) Å	θ = 2.5–26.5°
α = 70.114 (1)°	µ = 1.58 mm^−^^1^
β = 88.6311 (10)°	*T* = 173 K
γ = 66.765 (1)°	Prism, blue
*V* = 709.87 (3) Å^3^	0.50 × 0.50 × 0.45 mm

### Data collection

**Table:**

Bruker APEXII CCD diffractometer	2711 reflections with *I* > 2σ(*I*)
φ and ω scans	*R*_int_ = 0.025
Absorption correction: multi-scan (*SADABS*; Bruker, 2003)	θ_max_ = 26.6°, θ_min_ = 2.5°
*T*_min_ = 0.505, *T*_max_ = 0.536	*h* = −8→11
8672 measured reflections	*k* = −11→11
2945 independent reflections	*l* = −13→13

### Refinement

**Table:**

Refinement on *F*^2^	0 restraints
Least-squares matrix: full	Hydrogen site location: mixed
*R*[*F*^2^ > 2σ(*F*^2^)] = 0.027	H atoms treated by a mixture of independent and constrained refinement
*wR*(*F*^2^) = 0.073	*w* = 1/[σ^2^(*F*_o_^2^) + (0.0387*P*)^2^ + 0.4452*P*] where *P* = (*F*_o_^2^ + 2*F*_c_^2^)/3
*S* = 1.07	(Δ/σ)_max_ < 0.001
2945 reflections	Δρ_max_ = 0.26 e Å^−^^3^
233 parameters	Δρ_min_ = −0.57 e Å^−^^3^

### Special details

**Table:**

Geometry. All e.s.d.'s (except the e.s.d. in the dihedral angle between two l.s. planes) are estimated using the full covariance matrix. The cell e.s.d.'s are taken into account individually in the estimation of e.s.d.'s in distances, angles and torsion angles; correlations between e.s.d.'s in cell parameters are only used when they are defined by crystal symmetry. An approximate (isotropic) treatment of cell e.s.d.'s is used for estimating e.s.d.'s involving l.s. planes.

### Fractional atomic coordinates and isotropic or equivalent isotropic displacement parameters (Å^2^)

**Table:**

	*x*	*y*	*z*	*U*_iso_*/*U*_eq_	
Cu1	0.11824 (3)	0.26915 (3)	0.15525 (2)	0.01617 (9)	
N1	−0.2107 (2)	0.3725 (2)	0.34951 (17)	0.0208 (4)	
N2	−0.0714 (2)	0.3636 (2)	0.01433 (17)	0.0166 (3)	
N3	−0.3010 (2)	0.3463 (2)	−0.04965 (17)	0.0185 (3)	
N4	0.0614 (2)	0.0562 (2)	0.20396 (16)	0.0169 (3)	
N5	0.1657 (2)	0.4800 (2)	0.08348 (17)	0.0169 (3)	
N6	0.3708 (2)	0.2657 (2)	0.34195 (17)	0.0184 (3)	
N7	0.2554 (2)	0.2013 (2)	0.33410 (17)	0.0185 (3)	
N8	0.3280 (2)	0.1530 (3)	0.55105 (19)	0.0291 (4)	
O1	−0.1224 (2)	0.4373 (2)	0.27750 (19)	0.0352 (4)	
O2	−0.34096 (19)	0.4721 (2)	0.38151 (16)	0.0281 (3)	
O3	−0.1751 (2)	0.2133 (2)	0.38786 (18)	0.0361 (4)	
O4	0.3491 (2)	0.1266 (2)	0.07991 (19)	0.0289 (4)	
H41O	0.388 (3)	0.033 (4)	0.104 (3)	0.029 (8)*	
H42O	0.376 (3)	0.168 (4)	0.010 (3)	0.032 (8)*	
O5	0.5124 (2)	0.7700 (2)	0.15015 (19)	0.0254 (3)	
H51O	0.563 (4)	0.698 (4)	0.219 (3)	0.042 (9)*	
H52O	0.458 (4)	0.736 (4)	0.125 (3)	0.041 (9)*	
C1	−0.1560 (2)	0.2649 (2)	0.03218 (19)	0.0165 (4)	
C2	−0.0792 (2)	0.0862 (2)	0.13281 (19)	0.0172 (4)	
C3	0.1479 (3)	−0.1054 (3)	0.2939 (2)	0.0207 (4)	
H3	0.2474	−0.1283	0.3438	0.025*	
C4	0.0969 (3)	−0.2412 (3)	0.3170 (2)	0.0237 (4)	
H4	0.1614	−0.3551	0.3810	0.028*	
C5	−0.0481 (3)	−0.2088 (3)	0.2461 (2)	0.0239 (4)	
H5	−0.0858	−0.2994	0.2614	0.029*	
C6	−0.1384 (3)	−0.0411 (3)	0.1517 (2)	0.0221 (4)	
H6	−0.2389	−0.0149	0.1013	0.027*	
C7	0.3006 (2)	0.4957 (2)	0.1186 (2)	0.0173 (4)	
C8	0.4370 (2)	0.3469 (3)	0.2234 (2)	0.0204 (4)	
H8A	0.5019	0.2577	0.1842	0.025*	
H8B	0.5126	0.3913	0.2508	0.025*	
C9	0.2342 (3)	0.1361 (3)	0.4624 (2)	0.0227 (4)	
H9	0.1591	0.0820	0.4898	0.027*	
C10	0.4109 (3)	0.2351 (3)	0.4713 (2)	0.0249 (4)	
H10	0.4888	0.2679	0.5026	0.030*	

### Atomic displacement parameters (Å^2^)

**Table:**

	*U*^11^	*U*^22^	*U*^33^	*U*^12^	*U*^13^	*U*^23^
Cu1	0.01744 (14)	0.01210 (13)	0.01794 (14)	−0.00721 (9)	−0.00253 (9)	−0.00253 (10)
N1	0.0217 (9)	0.0206 (8)	0.0188 (8)	−0.0079 (7)	−0.0028 (7)	−0.0063 (7)
N2	0.0175 (8)	0.0123 (7)	0.0186 (8)	−0.0065 (6)	−0.0017 (6)	−0.0031 (6)
N3	0.0192 (8)	0.0183 (8)	0.0192 (8)	−0.0098 (7)	0.0007 (7)	−0.0052 (7)
N4	0.0192 (8)	0.0149 (7)	0.0165 (8)	−0.0071 (6)	0.0009 (6)	−0.0054 (6)
N5	0.0165 (8)	0.0132 (7)	0.0183 (8)	−0.0058 (6)	−0.0029 (6)	−0.0027 (6)
N6	0.0178 (8)	0.0149 (7)	0.0209 (9)	−0.0071 (6)	−0.0037 (6)	−0.0038 (7)
N7	0.0168 (8)	0.0169 (8)	0.0211 (9)	−0.0079 (6)	−0.0007 (6)	−0.0047 (7)
N8	0.0331 (10)	0.0328 (10)	0.0214 (9)	−0.0139 (8)	0.0005 (8)	−0.0090 (8)
O1	0.0337 (9)	0.0291 (8)	0.0463 (11)	−0.0184 (7)	0.0147 (8)	−0.0121 (8)
O2	0.0240 (8)	0.0294 (8)	0.0283 (8)	−0.0063 (6)	0.0038 (6)	−0.0128 (7)
O3	0.0486 (11)	0.0177 (8)	0.0398 (10)	−0.0139 (7)	0.0086 (8)	−0.0074 (7)
O4	0.0316 (9)	0.0164 (8)	0.0332 (10)	−0.0058 (7)	0.0129 (7)	−0.0079 (7)
O5	0.0245 (8)	0.0180 (7)	0.0312 (9)	−0.0098 (7)	−0.0023 (7)	−0.0045 (7)
C1	0.0187 (9)	0.0154 (9)	0.0170 (9)	−0.0089 (7)	0.0013 (7)	−0.0053 (7)
C2	0.0197 (9)	0.0165 (9)	0.0164 (9)	−0.0077 (7)	0.0031 (7)	−0.0067 (8)
C3	0.0219 (10)	0.0172 (9)	0.0195 (10)	−0.0059 (8)	0.0000 (8)	−0.0049 (8)
C4	0.0341 (12)	0.0143 (9)	0.0185 (10)	−0.0087 (8)	0.0023 (8)	−0.0025 (8)
C5	0.0361 (12)	0.0179 (9)	0.0226 (11)	−0.0162 (9)	0.0069 (9)	−0.0073 (8)
C6	0.0265 (11)	0.0212 (10)	0.0221 (10)	−0.0140 (8)	0.0015 (8)	−0.0069 (8)
C7	0.0167 (9)	0.0177 (9)	0.0179 (10)	−0.0082 (7)	0.0007 (7)	−0.0056 (8)
C8	0.0168 (9)	0.0196 (9)	0.0225 (10)	−0.0090 (8)	−0.0020 (8)	−0.0027 (8)
C9	0.0244 (10)	0.0204 (10)	0.0214 (10)	−0.0087 (8)	0.0019 (8)	−0.0058 (8)
C10	0.0281 (11)	0.0234 (10)	0.0226 (11)	−0.0101 (9)	−0.0038 (8)	−0.0079 (9)

### Geometric parameters (Å, º)

**Table:**

Cu1—N5	1.9709 (15)	N8—C9	1.349 (3)
Cu1—N2	1.9732 (16)	O4—H41O	0.71 (3)
Cu1—N4	2.0386 (16)	O4—H42O	0.79 (3)
Cu1—N7	2.0409 (17)	O5—H51O	0.78 (3)
Cu1—O4	2.2293 (16)	O5—H52O	0.76 (3)
N1—O3	1.234 (2)	C1—C2	1.463 (3)
N1—O1	1.244 (2)	C2—C6	1.376 (3)
N1—O2	1.266 (2)	C3—C4	1.391 (3)
N2—C1	1.326 (2)	C3—H3	0.9500
N2—N5^i^	1.356 (2)	C4—C5	1.377 (3)
N3—C7^i^	1.342 (2)	C4—H4	0.9500
N3—C1	1.346 (3)	C5—C6	1.391 (3)
N4—C3	1.335 (3)	C5—H5	0.9500
N4—C2	1.352 (3)	C6—H6	0.9500
N5—C7	1.329 (2)	C7—N3^i^	1.342 (2)
N5—N2^i^	1.356 (2)	C7—C8	1.496 (3)
N6—C10	1.328 (3)	C8—H8A	0.9900
N6—N7	1.368 (2)	C8—H8B	0.9900
N6—C8	1.455 (3)	C9—H9	0.9500
N7—C9	1.321 (3)	C10—H10	0.9500
N8—C10	1.323 (3)		
			
N5—Cu1—N2	93.75 (6)	N2—C1—N3	113.40 (17)
N5—Cu1—N4	172.58 (6)	N2—C1—C2	116.86 (17)
N2—Cu1—N4	80.29 (6)	N3—C1—C2	129.72 (17)
N5—Cu1—N7	88.59 (6)	N4—C2—C6	122.81 (18)
N2—Cu1—N7	161.96 (7)	N4—C2—C1	112.59 (16)
N4—Cu1—N7	98.45 (6)	C6—C2—C1	124.57 (18)
N5—Cu1—O4	87.79 (7)	N4—C3—C4	122.16 (19)
N2—Cu1—O4	108.72 (7)	N4—C3—H3	118.9
N4—Cu1—O4	89.95 (6)	C4—C3—H3	118.9
N7—Cu1—O4	89.23 (7)	C5—C4—C3	119.35 (19)
O3—N1—O1	120.96 (18)	C5—C4—H4	120.3
O3—N1—O2	119.57 (17)	C3—C4—H4	120.3
O1—N1—O2	119.45 (17)	C4—C5—C6	118.81 (18)
C1—N2—N5^i^	105.92 (15)	C4—C5—H5	120.6
C1—N2—Cu1	114.60 (13)	C6—C5—H5	120.6
N5^i^—N2—Cu1	137.73 (12)	C2—C6—C5	118.65 (19)
C7^i^—N3—C1	101.45 (15)	C2—C6—H6	120.7
C3—N4—C2	118.20 (16)	C5—C6—H6	120.7
C3—N4—Cu1	127.56 (14)	N5—C7—N3^i^	113.47 (17)
C2—N4—Cu1	114.23 (13)	N5—C7—C8	121.54 (17)
C7—N5—N2^i^	105.75 (15)	N3^i^—C7—C8	124.98 (17)
C7—N5—Cu1	126.66 (13)	N6—C8—C7	111.03 (16)
N2^i^—N5—Cu1	127.56 (12)	N6—C8—H8A	109.4
C10—N6—N7	108.72 (16)	C7—C8—H8A	109.4
C10—N6—C8	128.56 (17)	N6—C8—H8B	109.4
N7—N6—C8	122.68 (16)	C7—C8—H8B	109.4
C9—N7—N6	102.89 (16)	H8A—C8—H8B	108.0
C9—N7—Cu1	132.81 (14)	N7—C9—N8	114.40 (19)
N6—N7—Cu1	122.03 (12)	N7—C9—H9	122.8
C10—N8—C9	102.87 (18)	N8—C9—H9	122.8
Cu1—O4—H41O	121 (2)	N8—C10—N6	111.12 (18)
Cu1—O4—H42O	123 (2)	N8—C10—H10	124.4
H41O—O4—H42O	112 (3)	N6—C10—H10	124.4
H51O—O5—H52O	107 (3)		
			
C10—N6—N7—C9	0.2 (2)	N4—C3—C4—C5	−0.6 (3)
C8—N6—N7—C9	178.21 (17)	C3—C4—C5—C6	0.9 (3)
C10—N6—N7—Cu1	165.14 (14)	N4—C2—C6—C5	−1.3 (3)
C8—N6—N7—Cu1	−16.9 (2)	C1—C2—C6—C5	176.53 (18)
N5^i^—N2—C1—N3	0.8 (2)	C4—C5—C6—C2	0.0 (3)
Cu1—N2—C1—N3	168.39 (13)	N2^i^—N5—C7—N3^i^	0.0 (2)
N5^i^—N2—C1—C2	179.30 (16)	Cu1—N5—C7—N3^i^	178.11 (12)
Cu1—N2—C1—C2	−13.1 (2)	N2^i^—N5—C7—C8	−178.85 (17)
C7^i^—N3—C1—N2	−0.8 (2)	Cu1—N5—C7—C8	−0.8 (3)
C7^i^—N3—C1—C2	−179.06 (19)	C10—N6—C8—C7	−127.1 (2)
C3—N4—C2—C6	1.6 (3)	N7—N6—C8—C7	55.3 (2)
Cu1—N4—C2—C6	−179.68 (15)	N5—C7—C8—N6	−46.0 (2)
C3—N4—C2—C1	−176.44 (16)	N3^i^—C7—C8—N6	135.26 (19)
Cu1—N4—C2—C1	2.2 (2)	N6—N7—C9—N8	−0.5 (2)
N2—C1—C2—N4	7.0 (2)	Cu1—N7—C9—N8	−162.96 (15)
N3—C1—C2—N4	−174.73 (18)	C10—N8—C9—N7	0.5 (2)
N2—C1—C2—C6	−171.04 (18)	C9—N8—C10—N6	−0.3 (2)
N3—C1—C2—C6	7.2 (3)	N7—N6—C10—N8	0.1 (2)
C2—N4—C3—C4	−0.7 (3)	C8—N6—C10—N8	−177.76 (18)
Cu1—N4—C3—C4	−179.13 (14)		

Symmetry code: (i) −*x*, −*y*+1, −*z*.

### Hydrogen-bond geometry (Å, º)

**Table:**

*D*—H···*A*	*D*—H	H···*A*	*D*···*A*	*D*—H···*A*
O4—H41*O*···O5^ii^	0.71 (3)	2.03 (3)	2.735 (2)	172 (3)
O4—H42*O*···O5^iii^	0.79 (3)	1.96 (3)	2.735 (2)	168 (3)
O5—H51*O*···O2^iv^	0.78 (3)	2.02 (3)	2.773 (2)	163 (3)
O5—H52*O*···N3^i^	0.76 (3)	2.08 (3)	2.836 (2)	177 (3)
C5—H5···O1^ii^	0.95	2.43	3.360 (3)	166
C8—H8*A*···O4	0.99	2.56	3.160 (3)	119
C8—H8*B*···O2^iv^	0.99	2.36	3.319 (3)	162
C9—H9···O3^v^	0.95	2.44	3.205 (3)	137

Symmetry codes: (i) −*x*, −*y*+1, −*z*; (ii) *x*, *y*−1, *z*; (iii) −*x*+1, −*y*+1, −*z*; (iv) *x*+1, *y*, *z*; (v) −*x*, −*y*, −*z*+1.

## References

[bb1] Blake, A. J., Champness, N. R., Hubberstey, P., Li, W. S., Withersby, M. A. & Schröder, M. (1999). *Coord. Chem. Rev.* **183**, 117–138.

[bb2] Bruker (2003). *APEX2*, *SAINT* and *SADABS*. Bruker AXS Inc., Madison, Wisconsin, USA.

[bb3] Groom, C. R. & Allen, F. H. (2014). *Angew. Chem. Int. Ed.* **53**, 662–671. 10.1002/anie.201306438 24382699

[bb4] Gusev, A. N., Nemec, I., Herchel, R., Bayjyyev, E., Nyshchimenko, G. A., Alexandrov, G. G., Eremenko, I. L., Trávníček, Z., Hasegawa, M. & Linert, W. (2014). *Dalton Trans.* **43**, 7153–7165. 10.1039/c4dt00462k 24671486

[bb5] Haasnoot, J. G. (2000). *Coord. Chem. Rev.* **200–202**, 131–185.

[bb6] Khomenko, D. N., Doroshchuk, R. A., Egorov, O. A. & Lampeka, R. D. (2012). *Ukr. Khim. Zh.* **78**, 22–27.

[bb7] Klingele, J., Dechert, S. & Meyer, F. (2009). *Coord. Chem. Rev.* **253**, 2698–2741.

[bb8] Lin, W.-Q., Liao, X.-F., Jia, J.-H., Leng, J.-D., Liu, J.-L., Guo, F.-S. & Tong, M.-L. (2013). *Chem. Eur. J.* **19**, 12254–12258. 10.1002/chem.201301397 23794500

[bb9] Moliner, N., Gaspar, A. B., Muñoz, M. C., Niel, V., Cano, J. & Real, J. A. (2001). *Inorg. Chem.* **40**, 3986–3991. 10.1021/ic0100976 11466058

[bb10] Prins, R., Birker, P. J. M. W. L., Haasnoot, J. G., Verschoor, G. C. & Reedijk, J. (1985). *Inorg. Chem.* **24**, 4128–4133.

[bb11] Selmeczi, K., Réglier, M., Giorgi, M. & Speier, G. (2003). *Coord. Chem. Rev.* **245**, 191–201.

[bb12] Sheldrick, G. M. (2008). *Acta Cryst.* A**64**, 112–122. 10.1107/S0108767307043930 18156677

[bb13] Sheldrick, G. M. (2015). *Acta Cryst.* C**71**, 3–8.

[bb14] Soghomonian, V., Chen, Q., Haushalter, R. C., Zubieta, J. & O’Connor, C. J. (1993). *Science*, **259**, 1596–1599. 10.1126/science.259.5101.1596 17733025

[bb15] Zhou, Y.-H., Wan, W.-Q., Sun, D.-L., Tao, J., Zhang, L. & Wei, X. (2014). *Z. Anorg. Allg. Chem.* **640**, 249–253.

